# Composition of Commercial Licorice Candies and Development of Sugar-Reduced Licorice-Type Extruded Soft Candy Using Resistant Dextrin

**DOI:** 10.1021/acsomega.5c02581

**Published:** 2025-07-11

**Authors:** Funda Keskin Kuzey, Atefeh Karimidastjerd, Omer Said Toker, Muhammet Arici, Ibrahim Palabiyik, Murat Tasan, Nevzat Konar

**Affiliations:** † Halavet Food, Tuzla 34956, Istanbul, Turkey; ‡ 52999 Yildiz Technical University, Chemical and Metallurgical Engineering Faculty, Food Engineering Department, 34210 Istanbul, Turkey; § 162334 Namik Kemal University, Agricultural Faculty, Food Engineering Department, 59030 Tekirdağ, Turkey; ∥ 37504 Ankara University, Faculty of Agriculture, Department of Dairy Technology, 6170 Ankara, Turkey

## Abstract

Candy licorice is a confection consisting mainly of wheat flour and sugar syrup. In this study, resistant dextrin was used as a sugar substitute due to its beneficial properties, including fiber content, low sugar content, and low caloric value. In the first part of the study, the profiles of monosaccharides (glucose, fructose) and disaccharides (sucrose, maltose, and lactose) as well as the amounts of dietary fiber, total sugar, ash, crude protein, moisture, and fat (saturated, unsaturated, MUFA, and PUFA) were determined in different products (*n* = 5). In the second part, an optimization study was performed on licorice-type extruded soft sugar by adding resistant dextrin. The response variables for optimization included color (L*, b*, and a*), textural properties (hardness and chewiness), physicochemical properties (pH, water activity (*a*_w_), and total soluble solids), and sensory attributes (taste, structure, and general acceptability). The obtained and validated optimum composition was found for sucrose, resistant dextrin, and wheat flour as 15.0 g/100 g, 7.49 g/100 g, and 32.51 g/100 g, respectively. The *R* ^2^ values of models determined for these variables were between 0.739 and 0.999. Significant models were determined for pH, *a*_w_, L*, hardness, chewiness, and sensory properties (structure and general acceptability) (*p* < 0.05). The L*, a*, and b* values for the optimized candy sample with Brix, pH, and *a*_w_ values of 86.50, 2.83, and 0.6056, respectively, were 36.415, 17.798, and 3.262, respectively. The hardness of the sample was measured at 1588 g, while the chewiness value was 246.14. Hardness and chewiness values increased due to the amounts of wheat flour and resistant dextrin. Sensory analysis was conducted using a 9-point hedonic scale, evaluating taste, texture, and overall acceptability. The optimized sample received a score of 7.00 for taste, 7.20 for texture, and 7.30 for overall acceptability. Based on our findings, resistant dextrin can serve as an effective sucrose alternative and bulking agent in the development of low-calorie licorice, offering optimized texture, physicochemical characteristics, and sensory qualities.

## Graphical abstract

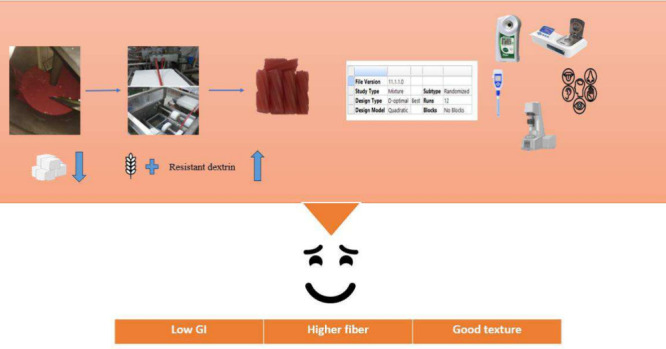

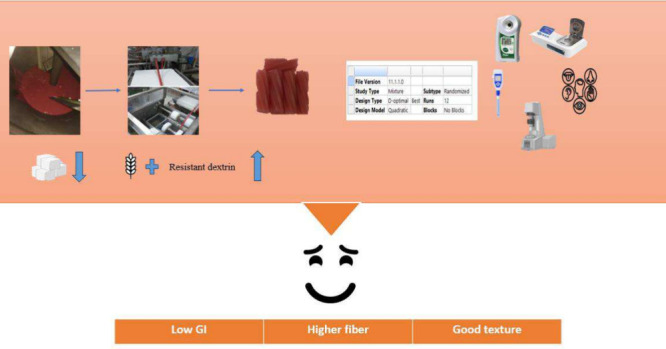

## 1 Introduction

In 2024, the global confectionery market was valued at $586.30 billion US dollars, with an expected annual growth rate of 5.33% (CAGR 2024–2028).^1^ Despite its popularity, licorice remains an under-researched confectionery category.^2^ Licorice-type extruded soft candy is produced by first mixing ingredients such as wheat flour, glucose syrup, sugar, oil, acidifiers, and starch and then cooking the dough under vacuum at 125–145 °C.^3^ Unlike other soft candies, licorice contains a significant amount of wheat flour (25–40%), which influences its structure, gelation, and caloric value. The flour’s starch composition, roughly 25% amylose and 75% amylopectin, affects gelation and texture. While gluten contributes to texture, starch gelation plays a more critical role.^4^ Thus, when developing lower-calorie licorice, the functional role of wheat flour must be carefully considered. The widespread use of sucrose and corn syrups (e.g., glucose syrups) in confectionery has driven increasing interest in alternative sweeteners.^5,6^ A major challenge in confectionery technology today is formulating low-calorie products without significantly compromising stability or quality.^5^ Additionally, guidance from international health authorities continues to influence consumer expectations and preferences. To effectively meet these demands, research must focus on sustainable and standardized alternative ingredients.^2^ Sugar reduction in confectionery can be achieved by partially replacing sucrose or glucose syrup with various alternative sweeteners and bulking agents. Alternatives like polyols (e.g., maltitol, isomalt), high-intensity sweeteners (e.g., stevia), and rare sugars (e.g., d-allulose) have been used in soft candies,^7,8^ as well as fruits and their byproducts.^9–15^ However, the use of grains^16^ and vegetables^17^ remains limited. Resistant dextrin (RD), a starch-based low-calorie ingredient derived from grains, such as corn or wheat, has shown promise in gummies. It offers nutritional benefits such as ∼70% fiber content, a low glycemic index, and low caloric value (2.10 kcal/g). RD contains multiple glycosidic bonds and is classified as generally recognized as safe (GRAS) by the FDA, with a safe daily intake of up to 45 g.^16,18^ Studies have shown that RD supports the growth of beneficial gut bacteria, adding a functional health benefit to confectionery products as a prebiotic.^19^ RD, a water-soluble dietary fiber, is made from starch by partial hydrolysis. Based on color, three types of RD can be identified as white dextrin, British gum, and yellow dextrin.^20^ Like other dietary fibers, it has some benefits including being an energy source for gut microbiota and reducing obesity, helping intestinal functions, and moderating the adsorption of sugar and neutral fats after meals. Therefore, it can be utilized in healthy snakes, dairy products, cakes, beverages, meat products,^20,21^ and also confectionery products.

Despite its widespread consumption, licorice remains an under-researched product in the confectionery sector. This study addresses that gap by first characterizing the proximate composition of various commercial licorice-type extruded soft candies, providing essential baseline data on existing market formulations. More importantly, it introduces a novel approach to developing a healthier licorice-type candy through the incorporation of resistant dextrin (RD), a starch-based, low-calorie, fiber-rich ingredient with proven prebiotic properties. While RD has been explored in gummies, beverages, and baked goods, its application in flour- and starch-rich licorice-type extruded candy has not been previously studied. This research uniquely evaluates RD’s functionality within this specific matrix, focusing on its impact on texture, gelation, and overall product quality when partially replacing sugar and flour. A Mixture Design approach was employed to systematically assess the effects of varying concentrations of sucrose, wheat flour, and RD on the physicochemical, color, texture, and sensory properties of the final product. The primary aim of the study was to explore the potential for sugar reduction in licorice-type extruded soft candy using RD and to determine the optimum formulation based on key quality parameters. By integrating compositional analysis with formulation optimization, this study provides both scientific insight and practical strategies for advancing low-calorie, fiber-enriched confectionery innovations.

## 2 Materials and Methods

### 2.1 Materials

DE 42 glucose syrup (80.0 ± 2.00 °Bx) (Cargill, Istanbul, Türkiye), palm oil (Cargill, Istanbul, Türkiye) as a surface coating material, sucrose (Keskinkılıç Gıda, Istanbul, Türkiye), wheat flour (Eris Un, Istanbul, Türkiye), distilled monoglyceride (Dupont Danisco–Dimodan, USA), citric acid (Tezkim Chemical, Istanbul, Türkiye), apple juice concentrate (70 °Bx), as an anthocyanin source black carrot concentrate (pH 3.05, >65.0 °Bx, total anthocyanin content >8.50 g/kg, db) (Döhler, Darmstadt, Germany), resistant dextrin (RD) (Nutriose FM10, Roquette, Lestrem, France), and strawberry flavor (Aromsa, Kocaeli, Türkiye) were used. Also, commercial licorice samples of various producers were obtained from the local market (Istanbul, Türkiye). The solubility, average molecular weight, dry substance, dietary fiber, and total mono- and disaccharide contents of RD were 60 g/100 g, 1100 g/mol, 95.0%, 70.0%, and <15.0%, respectively.^18^

### 2.2 Commercial Product Characterization

#### 2.2.1 Sampling

Samples with different brands and color characteristics were obtained from the local market (Istanbul, Türkiye) (*n* = 5). They were strawberry, blackberry, mixed fruit, apple, and tropical fruit-flavored products.

#### 2.2.2 Analysis

Various analytical studies were conducted to characterize the licorice candy samples. The elemental and mineral contents (iron, copper, calcium, arsenic, lead, and sodium) were analyzed using the method described by Babu et al.,^22^ which involved microwave sample digestion (Multiwave 7101, Anton-Paar, Austria) and inductively coupled plasma mass spectrometry (ICP-MS) (NexION 350 X, PerkinElmer, USA). Chromatographic analyses were performed on samples prepared using the method described by Vojvodić Cebin et al.^23^ to determine sugar profiles. The amounts of sucrose, glucose, fructose, lactose, and maltose were determined using an HPLC system (Agilent 1200, Agilent Technologies, Santa Clara, CA, USA) with a Zorbax Hi-PlexCa (7.7 × 300 mm) column and RI detector. Calibration curves are given in the Supporting Information. Total fat and total SFA, MUFA, and PUFA were determined using the method described by Trattner et al.^24^ For this aim, a GC instrument (Agilent 6890, Agilent Technologies, Santa Clara, CA, USA) with a flame ionization detector (FID) was used. The moisture content, crude protein, and dietary fiber content of the licorice confectionery samples were determined using the methods described by Gok et al.,^16^ AOAC 920.87, and AOAC 991.43,^25^ respectively. Proximate analysis results are given in Table 1.

**Table 1:** Proximate Composition, Sugar Profile, and Elemental and Mineral Contents of Commercial Licorice^a^ Soft Candy Samples

Composition	Mean ± Standard Deviation
Fructose (g/100 g)	8.79 ± 2.32
Glucose (g/100 g)	15.40 ± 3.70
Sucrose (g/100 g)	24.9 ± 10.9
Lactose (g/100 g)	nd
Maltose (g/100 g)	7.11 ± 0.20
Dietary fiber (g/100 g)	0.00 ± 0.00
Total sugar (g/100 g)	53.20 ± 4.60
Total ash (g/100 g)	0.21 ± 0.07
Moisture content (g/100 g)	8.15 ± 1.42
Crude protein (g/100 g)	0.91 ± 0.56
Total fat (g/100 g)	2.76 ± 0.05
Total saturated fatty acids (g/100 g)	1.35 ± 0.12
Total unsaturated fatty acids (g/100 g)	1.24 ± 0.11
Monounsaturated fatty acids (g/100 g)	0.93 ± 0.06
Polyunsaturated fatty acids (g/100 g)	0.31 ± 0.05
Iron (mg/kg)	9.66 ± 3.05
Copper (mg/kg)	nd
Calcium (mg/kg)	7.59 ± 1.59
Arsenic (mg/kg)	nd
Lead (mg/kg)	nd
Sodium (mg/100 g)	nd

a The number of commercial samples was five. nd: not determined. Results are expressed as mean of triplicate analyses.

### 2.3 Study Design and Sample Preparation

#### 2.3.1 Mixture Design

Considering sugar (sucrose) (10.0–30.0 g/100 g), resistant dextrin (RD) (0.00–20.0 g/100 g), and wheat flour (25.0–35.0 g/100 g) concentrations as independent variables, a study plan with 12 points was determined by using Design Expert version 7, and the mixture model type was Quadratic (Table 2). All other process variables were maintained as constant. The ranges of the independent variables were determined based on preliminary studies conducted on industrial applications and product compositions, as well as previous studies involving the use of soluble wheat fiber.^16^ According to this design, extruded licorice candy samples were produced as 10 kg of dough for each formulation. For this purpose, a pilot coextruder (Exrufood, Kervan, Istanbul, Türkiye) was used. Also, a control sample to define responses for optimization was prepared using sucrose (24.2 g/100 g) and wheat flour (30.8 g/100 g).

**Table 2:** Extruded Soft Candy Optimization Independent and Dependent Compositional Variables (g/100 g)

Sample	Sucrose	Resistant Dextrin (RD)	Wheat Flour	Glucose Syrup (42DE)	Apple Juice Concentrate	Palm Oil	Distilled Monoglyceride	Citric Acid	Water	Colorant	Flavor
1	10.000	14.688	30.312	34.150	1.500	2.000	0.100	1.250	5.800	0.100	0.100
2	17.435	6.207	31.358	34.150	1.500	2.000	0.100	1.250	5.800	0.100	0.100
3	13.220	10.739	31.042	34.150	1.500	2.000	0.100	1.250	5.800	0.100	0.100
4	20.597	9.403	25.000	34.150	1.500	2.000	0.100	1.250	5.800	0.100	0.100
5	10.598	19.402	25.000	34.150	1.500	2.000	0.100	1.250	5.800	0.100	0.100
6	24.025	3.430	27.546	34.150	1.500	2.000	0.100	1.250	5.800	0.100	0.100
7	30.000	0.000	25.000	34.150	1.500	2.000	0.100	1.250	5.800	0.100	0.100
8	30.000	0.000	25.000	34.150	1.500	2.000	0.100	1.250	5.800	0.100	0.100
9	11.105	8.895	35.000	34.150	1.500	2.000	0.100	1.250	5.800	0.100	0.100
10	21.401	0.000	33.599	34.150	1.500	2.000	0.100	1.250	5.800	0.100	0.100
11	11.105	8.895	35.000	34.150	1.500	2.000	0.100	1.250	5.800	0.100	0.100
12	15.310	12.648	27.042	34.150	1.500	2.000	0.100	1.250	5.800	0.100	0.100

#### 2.3.2 Sample Preparation

Confectionery doughs were prepared according to the formulations provided in Table 3 by using a pilot-scale tank. All ingredients, except for color and flavor agents, were mixed in the tank at 50–55 °C and stirred at 15 rpm for 15–20 min until a homogeneous mixture was achieved. Once homogenized, black carrot concentrate (15 g per 10 kg of dough) was added for coloring. The colored dough was then transferred into the extruder, where it was cooked at 130 °C under vacuum conditions (0.8 bar). A twisted shape was formed as the final product. During extrusion, 0.25 g/100 g strawberry flavoring was added to the dough. Following extrusion, the samples were cooled to +4 °C using a water-cooling conveyor. The cooled samples were then coated with palm oil by the dipping method. After oiling, the samples were cut into 25–30 mm lengths using a guillotine knife and finally packaged.

**Table 3:** Physicochemical TSS (°Bx), pH, Water Activity (*a*_w_), and L*, a*, and b* Analysis Results of Extruded Soft Candy Samples^b^

Sample	TSS (°Bx)	pH	Water Activity (*a*_w_)	L*	a*	b*	Hardness (g)	Chewiness (g)
1	85.5 ± 0.21	2.95 ± 0.04	0.641 ± 0.001	35.2 ± 1.31	14.3 ± 1.10	2.86 ± 0.40	2626 ± 169	314 ± 37
2	87.0 ± 0.38	2.98 ± 0.01	0.626 ± 0.002	35.5 ± 1.03	15.4 ± 1.37	3.08 ± 0.55	1670 ± 102	250 ± 28
3	86.0 ± 0.31	2.96 ± 0.01	0.621 ± 0.002	36.7 ± 0.81	14.8 ± 0.47	2.52 ± 0.48	3446 ± 255	435 ± 47
4	86.0 ± 0.25	2.93 ± 0.02	0.596 ± 0.003	35.8 ± 0.85	14.1 ± 1.00	1.70 ± 0.20	1838 ± 110	148 ± 06
5	87.0 ± 0.47	3.04 ± 0.02	0.659 ± 0.003	32.8 ± 1.33	13.3 ± 0.81	2.66 ± 0.25	3763 ± 282	465 ± 44
6	85.5 ± 0.19	2.99 ± 0.02	0.612 ± 0.002	36.3 ± 0.53	15.3 ± 1.56	2.21 ± 0.71	1562 ± 53	196 ± 05
7	86.0 ± 0.29	2.97 ± 0.02	0.570 ± 0.002	34.1 ± 1.02	17.5 ± 2.36	2.69 ± 0.53	1323 ± 112	137 ± 15
8	86.0 ± 0.20	2.97 ± 0.01	0.569 ± 0.004	34.4 ± 0.77	15.2 ± 0.63	1.28 ± 0.43	1095 ± 42	127 ± 07
9	85.0 ± 0.22	2.93 ± 0.02	0.639 ± 0.002	36.1 ± 0.68	14.4 ± 1.33	2.38 ± 0.56	1789 ± 114	180 ± 13
10	86.5 ± 0.40	3.07 ± 0.02	0.611 ± 0.004	34.4 ± 0.81	16.1 ± 0.93	2.42 ± 0.34	2694 ± 185	375 ± 23
11	85.0 ± 0.26	2.93 ± 0.02	0.639 ± 0.002	36.0 ± 0.47	15.5 ± 0.74	3.19 ± 0.49	1855 ± 107	190 ± 20
12	86.0 ± 0.21	2.97 ± 0.01	0.597 ± 0.002	36.8 ± 1.60	16.4 ± 0.37	3.89 ± 0.23	2352 ± 185	224 ± 42
*p*-value	0.0972	0.0001^a^	0.0007^a^	0.0112^a^	0.7069	0.7434	0.0163^a^	0.0030^a^
*R* ^2^	0.9775	0.9837	0.9998	0.9975	0.7613	0.7392	0.9964	0.9993

a TSS: total soluble solids; mean ± standard deviation.

b The model was significant (*p* < 0.05) and *p*-value of lack of fit was higher than 0.05.

Figure 1 displays various extruded licorice-type soft candy samples, all produced under the same processing conditions, with varying amounts of the main ingredients, as specified in Table 2.

**Figure 1:**
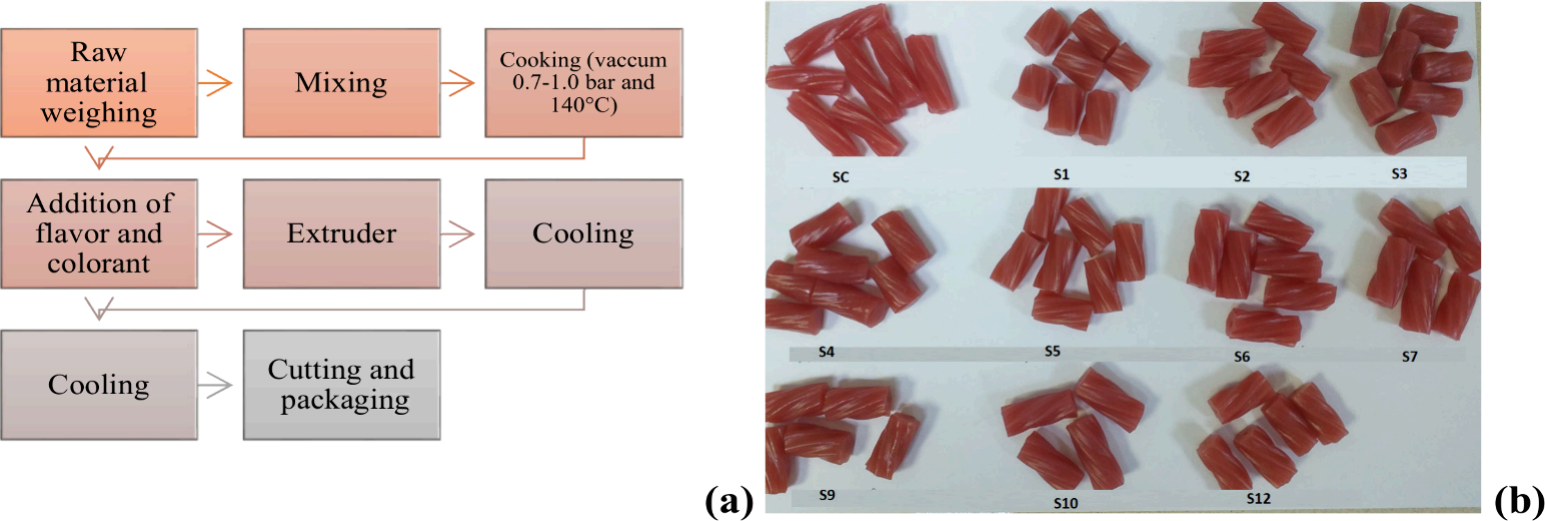
Production flowchart of licorice-type extruded soft candy (a) and control sample (SC) and sugar-reduced extruded licorice-type soft candy samples (b).

### 2.4 Total Soluble Solids

The total soluble solids (TSS) content of samples was determined by using a modified method described by Mir et al.^26^ For this purpose, 5 g of each sample was dissolved in 5 mL of distilled water at a scale of 1:1 and placed in a closed beaker. Melting was ensured by means of a magnetic heater stirrer (Weightlab WF-H380A) until a homogeneous mixture was obtained, taking care to avoid evaporation. The homogeneous liquid was then poured into the glass of a refractometer (Atago PAL-3 Digital Refractometer 0–93 total soluble solids), and the total soluble solids content was determined.

### 2.5 Water Activity

Water activity (*a*_w_) values of the samples were determined by using a water activity analyzer (Aqualab 4TE, Aqualab, USA); 2 g of each sample was placed in the device cabinet at 20 °C.^16^

### 2.6 pH

10 g of each sample was distilled with 90 mL of water, and the pH value of the solution was measured by a pH meter (S210, Mettler Toledo, USA). The probe was calibrated with standard buffer solutions before each measurement.^27^

### 2.7 Color Analysis

A colorimeter (CR-400 Konica, Minolta, Tokyo, Japan) was used for the color analysis of samples. For each sample, 5 parallel measurements were taken to determine L* (brightness), a* (±red-green), and b* (±yellow-blue) values.^16^

### 2.8 Texture Analysis

Texture analysis of horizontally placed samples (15.0 mm × 15.0 mm × 20.0 mm) was performed using a P/36 probe and a texture analyzer device (TA.HD Plus, Stable Micro Systems, Godalming, England) equipped with a 5 kg load cell operating at a constant speed of 1 mm/s. The time between the two compressions, each compressing the sample to 50% of its original height, was set to 15 s. From the resulting force–time curve, the textural properties of hardness (g) and chewiness (g) of the samples were determined. Analyses were performed in 5 parallel replicates.^16^

### 2.9 Sensory Evaluation

Sensory evaluation of the samples was carried out at the Kervan Food Center with 45 trained panelists, both male and female, aged between 28 and 45. The extruded soft candy samples were randomly coded and presented to the panelists. The samples were evaluated for taste, structure (including liking of firmness, elasticity, and chewiness), and general acceptability using a 9-point hedonic scale (1 = disliked very much, 9 = liked very much).^16,9^

### 2.10 Statistical Analysis

The results of the analyses conducted for all samples were determined as the mean ± standard deviation. Regression coefficients and interaction terms were determined. Lack of fit, regression, and *p*-values were used to verify models related to the effects of independent variables on licorice samples. For a significant model, lack of fit should be not significant. *p*-value was applied for the selected model and coefficients to study the significance of each parameter in the equation. A *p*-value of less than 0.05 indicates that model terms are significant. When a factor has a *p*-value smaller than 0.05, it influences independent variable(s) in a significant way for a confidence level of 0.95. If the *p*-value is greater than 0.05, the possibility of the existence of other coefficients in the equation is greater than 95% which means the provided parameter cannot be significant.^27^ To determine model validity, the experimental and predicted values were compared, and the results were found between 95% prediction intervals.

### 2.11 Ethics Statement

The sensorial trial was performed according to the European Directive 2010/63/UE and with the permission of Yildiz Technical University (Istanbul, Türkiye).

## 3 Results and Discussion

### 3.1 Licorice Soft Candy Composition

The proximate composition, sugar profile, and elemental and mineral contents of commercial licorice soft candy samples are listed in Table 1. The composition of confectionery products generally shows a wide range of variation.^5,28^ Among the primary reasons for this are the broad variations in formulation and technology depending on the manufacturer.^4^ A similar trend was observed in commercial licorice candies. Carbohydrates were the major components in all samples, with sucrose being the predominant sugar, aligning with the focus of this study. The results suggest that sucrose should primarily be considered as a main carbohydrate in licorice. However, the wide range of sucrose content in commercial samples is noteworthy (24.9 ± 10.9 g/100 g). Lactose was not detected in any of the samples. Similarly, the absence of lead, copper, and arsenic in the samples was a positive result from a food safety perspective. The protein content in licorice is believed to originate primarily from stabilizers, with wheat flour, commonly used as both a stabilizer and a gelling agent, being the main source.^4^ The fat content in licorice samples was negligible, with a balance between saturated and unsaturated fatty acids. It was found that the small amount of fat, intended to prevent moisture loss through surface coating applications, varied within a narrow range across all commercial samples. Licorice manufacturers particularly consider moisture content to control and/or regulate the textural properties of the product. Achieving a target moisture content of <15.0 g/100 g in these confectionery products, which also ensures sufficient water for proper starch gelatinization, requires careful control of the thermal processing conditions.^16^ In the commercial samples, the moisture content was generally determined to be below 10.0 g/100 g.

### 3.2 Physicochemical Properties

Total soluble solids (TSS), water activity, and pH as physicochemical properties of the samples are given in Table 3. TSS content of the control sample was measured at 86.0 ± 0.31, while the values for the other samples ranged from 85.0 to 87.0 (Table 3). According to the ANOVA results (Table 3), the model was not statistically significant (*p* > 0.05), indicating that replacing sucrose with RD did not have a notable effect on the TSS content of the samples. Figure 2a expresses a slight increase in TSS when both wheat flour and RD were used in higher amounts in the formulation. Elevated TSS value can lead to quality loss during shelf life.^28^ For all soft candy products, TSS (expressed in °Bx) is a critical parameter, as it significantly influences both the stability and the textural properties of the product.^22^ The TSS results confirm that replacing sucrose with RD can achieve TSS levels comparable to those of the control sample. Unlike native starch, RD achieves greater solubility when processed at higher temperature and acidic conditions during cooking and extruding.^29^ It has been shown that the increased solubility of resistant maltodextrins is due to starches being broken down into smaller molecules during pyrolytic processing. This process involves the hydrolysis of α-1,4 and α-1,6 glycosidic bonds by enzymes such as α-amylase and amyloglucosidase.^30^ The pH value affects gel formation, color, and also sucrose inversion (which influences the sweetness of product) in jelly/gummy soft candies.^28^ For licorice-type extruded soft candy, the recommended pH range is reported to be 2.80–3.50.^31^ A low pH value can cause problems such as melting in products during shelf life. In contrast, at high pH values, the gel structure may be inhibited during the cooking process, and loss of shape of the final products may occur.^16^ Moreover, pH is a critical factor in products fortified with pH sensitive bioactive components like antioxidants and anthocyanins, as it directly affects their stability and efficacy.^31^ The model *p*-value of 0.0001 was found to be significant for pH (*p* < 0.05) (Table 3). Additionally, Figure 2c illustrates the effects of the independent variables on the pH values of the samples. As shown in the graph, the pH values decreased with an increased amount of RD while the other variables were held constant. The effects of poly- and oligosaccharides varied on this parameter due to the used confectionery matrix. For instance, Hasani and Yazdanpanah^32^ determined that the replacement of pectin with gum had no effect on the pH of jelly samples. The use of materials with different hygroscopic properties can affect the pH values of foods due to their buffering capacity, impact on moisture content, and effects on solubility and ionic balance. In studies where RD was used to replace various components in dairy products, it was found to have negligible effects on the sample pH values.^33,34^ However, the use of dietary fibers in gels has been shown to influence pH values, with those of grain origin causing a reduction in these values.^35^

**Figure 2:**
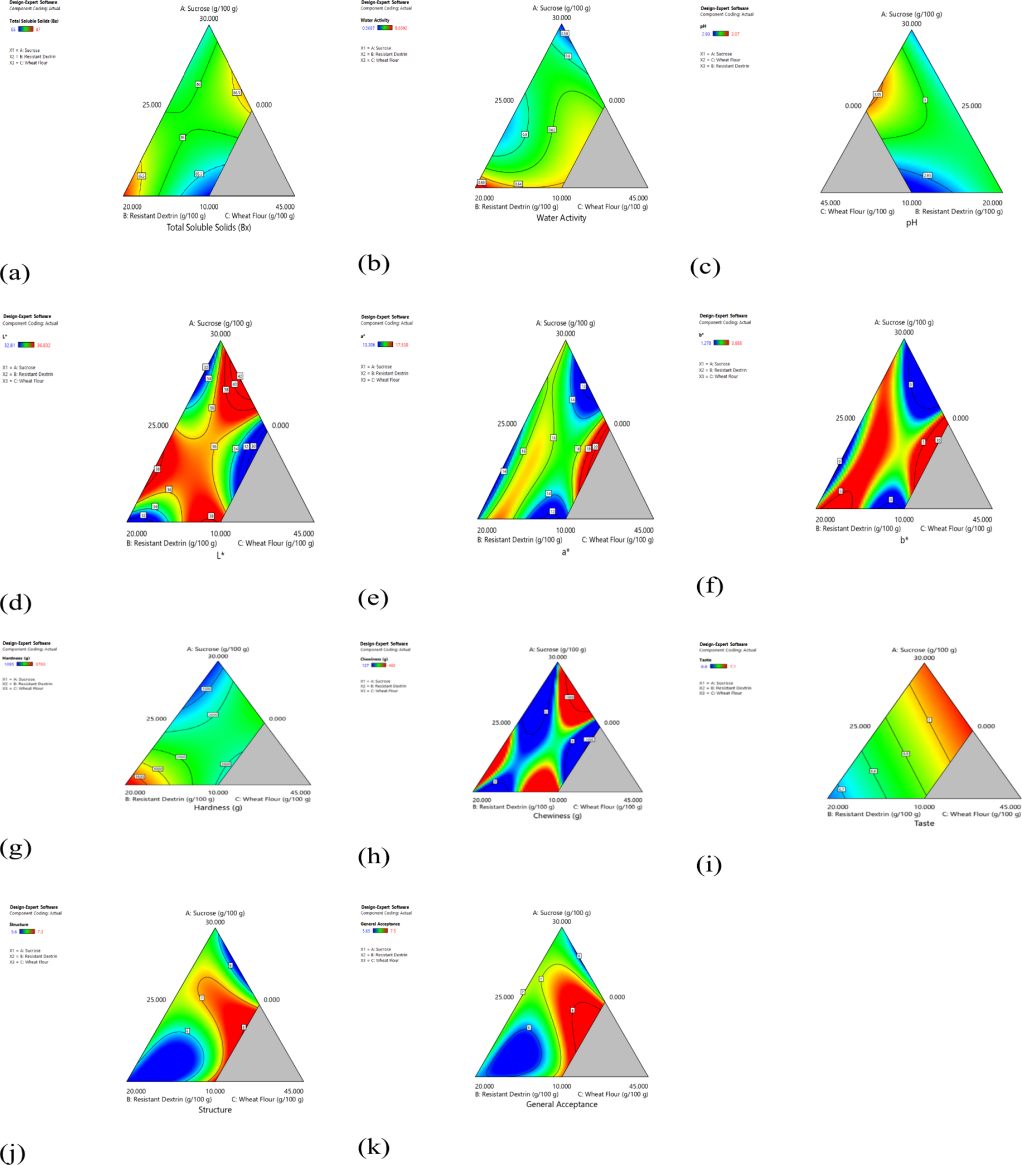
3D surface graphs of licorice-type extruded soft candy with ingredients A: sucrose, B: resistant dextrin, and C: wheat flour effects on properties: (a) total soluble solids, (b) *a*_w_, (c) pH, (d) color L* value, (e) color a* value, (f) color b* value, (g) hardness, (h) chewiness, (i) taste acceptability, (j) textural acceptability, and (k) general acceptability.

Water activity (*a*_w_) is a crucial criterion for soft candies, as the growth of yeast and molds is inhibited at *a*_w_ values of around 0.6.^36^ In this study, the model describing the effects of independent variables on *a*_w_ was statistically significant (*R* ^2^ = 0.9998, *p* < 0.05). As shown in Figure 2b, the *a*_w_ decreased with the reduction of resistant dextrin (RD). The lowest *a*_w_ value (0.5687) was observed in the sample containing 0% RD, 25% wheat flour, and 30% sucrose. In soft confectioneries, *a*_w_ can vary depending on the type and concentration of hydrocolloids used.^37^ In the case of licorice, the water content varies, generally 16–20% for licorice, while harder gums contain only 7–8%. During cooking, gelatinized starch and denatured proteins form a chewy matrix with sugars. These candies have a semisolid texture due to hydrocolloid cross-linking, which traps sugar syrup in a gel network.^4^ Consequently, it is expected that replacing wheat flour in the composition can influence *a*_w_. However, the molecular weight of the substitute material also plays a role. An increase in materials with a lower molecular weight can lead to a reduction in the glass transition temperature (*T*_g_) of the mixture. Changes in the *T*_g_ value of the matrix result in alterations in the water activity and mobility. In sugar–water mixtures, since water molecules are significantly smaller than sugars, they exhibit considerable mobility even at temperatures slightly below *T*_g_.^38^ The substitution of sucrose and/or wheat flour with RD, which contains less than 15% mono- and disaccharides, likely altered *a*_w_ due to these interactions.

Previous studies have reported that the *a*_w_ value of licorice products is typically in the range between 0.50 and 0.75.^31^ In agreement with these findings, the *a*_w_ values of the formulated samples in this study were found to be in safe range. In fact, *a*_w_ is dependent on the moisture affinity of the sugar substitutes used in the formulation. The water bounding ability or swelling of these components can affect the physicochemical and textural properties as well as the shelf life of candies.^28^

### 3.3 Color

Color is a critical quality parameter for products and must be carefully assessed.^9^ Black carrot concentrate, a natural colorant commonly used in confectionery technology, was utilized in the preparation of the samples. In interpreting the study findings, the inclusion of a natural colorant in the formulation and its interaction with the independent variables were taken into consideration. The results of the color analysis are presented in Table 3 and Figure 2. ANOVA analysis indicated that for L*, the *p*-value was 0.0112 (*p* < 0.05) with an *R* ^2^ of 0.9975. The L* values of the control sample (35.35) and the 12 formulated samples (32.810–36.832) suggest that the brightness levels were near those of the control sample. As shown in Figure 2d, an increase in the RD content resulted in lower L* values. The color of RD ranges from white to light yellow but may darken during production due to heat, acid concentration, and processing time, potentially affecting consumer acceptance. High substitution levels can lead to unwanted yellowing. Thus, controlling factors like acid type, concentration, and pyrolysis conditions is crucial to ensure desirable color and product quality.^29^ Additionally, sucrose had a more dominant effect on brightness compared to RD. For instance, when comparing samples 4 and 5, which both contained 25.000% wheat flour, the sample with higher sucrose and lower RD exhibited a higher L* value (35.768) compared to the other sample (32.810) (Table 3).

The model for the a* value (*R* ^2^ = 0.7613) was not statistically significant (*p* > 0.05). The control sample showed a value of 18.943, while all formulated samples exhibited reduced redness levels. Among them, samples 7, 10, and 12 were closest to the control, with a* values of 17.5, 16.1, and 16.4, respectively (Table 3). To achieve a* values close to the control while reducing sucrose content, the use of RD appears to be the key factor. As illustrated in Figure 2e, the sample without RD (0.000%) or reduced sucrose had the highest a* value, indicating the most intense redness. This finding may also be associated with the establishment of formulation conditions that enhance the process stability of the black carrot concentrate. For the b* value, the model showed that none of the components were significant factors (*p* > 0.05). The b* value of the control sample was 3.2, and the positive b* values of the other 12 samples suggest a shift toward yellowness, similar to the control. Although the model was not significant, Figure 2f indicates that increasing the content of dextrin and wheat flour led to a shift in the red region, causing more yellowness in the samples. Among all samples, sample 8 (30% sucrose, 0% resistant dextrin, 25% flour) exhibited the lowest b* value of 1.28 ± 0.43, indicating minimal yellowness, while sample 12, with sucrose reduced to 15.3% and RD increased to 12.6%, exhibited the highest b* value (3.89 ± 0.23), indicating increased yellowness. In related studies, a combination of stevia extract and 5% buttermilk powder was used to produce cookies with 30% and 60% reduced sugar content. The results demonstrated that this combination resulted in cookies with properties similar to those of the control.^39^ Mutlu et al.^40^ reported significant reductions in the L* value of soft jelly candies, while Gok et al.^16^ observed no significant effects (*p* > 0.05) of soluble wheat fiber concentration on the redness (a*) of gummy candies. Modern licorice formulations typically include sugar, glucose syrup, wheat flour, and vegetable oil, along with fruit flavors (e.g., strawberry, apple, lemon) and food colorings.^41,4^ Hydrocolloids, particularly agar and guar, significantly influence the color properties of fruit pastilles. Increased agar concentrations can enhance brightness and transparency while also intensifying red (a*) and yellow (b*) color indices due to Maillard reactions and pigment oxidation. Guar gum may protect β-carotene, contributing to an increased yellowness (b*). Different hydrocolloid levels can either promote or inhibit nonenzymatic browning and pigment-related changes in color.^42^ In future studies, the stability behavior and interactions of other colorants—beyond black carrot concentrate—within the licorice formulation should also be investigated. Consumer preferences are closely tied to the flavor and appearance of the product; for example, those choosing apple-flavored candies expect an apple-like taste, aroma, and a reddish color.^43^

### 3.4 Texture

Texture analysis results of the samples are given in Table 3. The effects of varying the amounts of sucrose, resistant dextrin (RD), and wheat flour components on the texture properties of the final product samples were examined based on hardness and chewiness parameters. According to ANOVA results, the model for hardness (*R* ^2^ = 0.9964, *p* < 0.05) was found to be significant, indicating that the three variable ingredients had a significant effect on the hardness of the licorice-type soft extruded candy. As shown in Figure 2g, the Mixture Design model illustrates that hardness increases with higher levels of RD, lower levels of sucrose, and increased amounts of wheat flour, particularly in the red regions of the plot. In general, it can be extracted that RD and flour are the ingredients that increased the hardness of the final product. For example, the highest hardness values were observed in samples 3 and 5 (Table 3). The hardness values recorded in this study were generally higher than those reported in the literature for jelly and gummy candies.^7,44^ Sample 5, containing 10.5 g/100 g sucrose, 19.4 g/100 g RD, and 25 g/100 g wheat flour, exhibited the highest hardness. In contrast, sample 8, formulated with 30 g/100 g sucrose, 0 g/100 g RD, and 25 g/100 g wheat flour, showed the lowest hardness.

Chewiness is considered an important texture property for gummy and jelly candies, as it plays a key role in determining elasticity.^44^ Figure 2h illustrates that the chewiness of the samples increases with higher amounts of RD. Chewiness followed a trend similar to that of hardness, with sample 8 showing the lowest value (127 ± 07) and sample 5 the highest (465 ± 44), indicating a positive correlation between these two textural attributes. Additionally, in samples 7, 8, and 10, which all contain the same amount of RD (0%) (Table 3), an increase in wheat flour from 25% to 30% further enhances the chewiness. Consequently, RD and wheat flour were identified as the most influential factors affecting chewiness. The overlap between the chewiness and hardness results can be considered as validation of the model’s accuracy. In a study on fruity candies, agar was used to substitute sucrose and glucose syrup. These sweeteners were replaced with polydextrose, oligofructose, sucralose, and erythritol. Results indicated that acidity and stickiness values were lower in the sugar-reduced samples. On the other side, sensory analysis showed that sugar-reduced candies met consumer expectations and could offer additional health benefits.^45^ In another study, gelatin-based soft candy was formulated with inulin as a substitute for corn starch. The sample with 9% inulin showed values similar to those of the reference sample in terms of hardness, sickness, chewiness, flexibility, and gelling properties. The textural properties of inulin-containing candies were found to be similar to those of the reference. Inulin as a resistant dietary fiber was found to be a promising substitute.^46^ The addition of agar, especially in combination with licorice (glycyrrhetinic acid), was reported to enhance the textural firmness by increasing the proportion of soluble solids. The highest quantities of agar and licorice also caused the highest chewiness due to their interaction effect.^42^ There are currently no reported applications of RD in confectionery. However, its successful use in pasta and flour-based products suggests the potential for increasing dietary fiber without compromising texture or palatability. RD exhibits plasticizing properties similar to those of sucrose, supporting desirable dough and baking qualities while maintaining sensory acceptance. This highlights RD’s promise in developing healthier foods aimed at addressing obesity and diabetes.^29^ For example, biscuits enriched with RD showed the highest firmness (1609 ± 388 g) compared with the control and other samples. RD increased dough stickiness and liquidity due to its low molecular weight and amorphous structure, which allows its free hydroxyl groups to strongly bind with water, reflecting its high hygroscopicity.^47^

### 3.5 Sensory Properties

The influence of varying sucrose, resistant dextrin (RD), and wheat flour levels on sensory attributes—taste, texture, and overall acceptability—was evaluated using a structured 9-point hedonic scale (Table 4; Figure 2i–k). Analysis of variance (ANOVA) was conducted for each sensory parameter, followed by Tukey’s Honestly Significant Difference (HSD) post-hoc test to identify significant differences among formulation means (α = 0.05). Confidence intervals (95%) were also calculated to assess the precision of mean scores. The regression model for taste did not reach statistical significance (*p* > 0.05), indicating that variations in sucrose, RD, and wheat flour levels did not significantly predict taste scores within the tested range. However, models for texture and overall acceptability were statistically significant (*p* < 0.05), with high coefficients of determination (*R* ^2^ = 0.9712 for taste, 0.9900 for structure, and 0.9947 for general acceptability), suggesting strong model fits. As illustrated in Figure 2i, although the taste model was not significant, sensory scores consistently increased with higher sucrose levels. The highest taste score (mean = 7.1, 95% CI: 6.8–7.4) was observed at 30% sucrose concentration (Table 4). Tukey’s HSD test revealed that this formulation was significantly preferred over those with lower sucrose levels (*p* < 0.05), reinforcing the sensory panel’s consistent preference despite the lack of significance in the regression model. This indicates that sucrose concentration had a perceptible, though not statistically modeled, impact on taste perception under the given experimental conditions.

**Table 4:** Sensory Evaluation Results of Formulated Extruded Soft Candies^a^

Sample	Sucrose (g/100 g)	Resistant Dextrin (RD) (g/100 g)	Wheat Flour (g/100 g)	Taste	Structure	General Acceptability
1	10.000	14.688	30.312	6.90 ± 1.10 b	5.70 ± 2.30 f	6.15 ± 0.85 d
2	17.435	6.207	31.358	7.00 ± 0.50 a	7.00 ± 1.00 b	7.50 ± 0.50 a
3	13.220	10.739	31.042	6.90 ± 1.10 b	5.60 ± 1.40 f	6.00 ± 1.00 d
4	20.597	9.403	25.000	6.90 ± 2.10 b	6.75 ± 1.25 c	7.00 ± 1.00 b
5	10.598	19.402	25.000	6.60 ± 1.40 d	5.70 ± 1.30 f	6.25 ± 0.75 c
6	24.025	3.430	27.546	7.05 ± 0.95 a	6.90 ± 1.10 b	7.15 ± 0.85 b
7	30.000	0.000	25.000	7.00 ± 1.00 a	6.20 ± 1.80 d	6.45 ± 1.55 c
8	30.000	0.000	25.000	7.10 ± 0.90 a	6.45 ± 1.55 d	6.45 ± 0.55 c
9	11.105	8.895	35.000	6.90 ± 1.10 b	7.15 ± 0.85 a	7.30 ± 0.70 a
10	21.401	0.000	33.599	7.05 ± 0.95 a	6.05 ± 0.95 e	6.55 ± 0.45 c
11	11.105	8.895	35.000	6.85 ± 1.15 b	7.30 ± 0.70 a	7.50 ± 0.50 a
12	15.310	12.648	27.042	6.75 ± 1.25 c	5.80 ± 1.20 f	5.85 ± 1.15 e
*p*-value	-	-	-	0.1234	0.0443^b^	0.0235^b^
*R* ^2^	-	-	-	0.9712	0.9900	0.9947

a Mean ± standard deviation.

b The model was significant (*p* < 0.05) and *p*-value of lack of fit was higher than 0.05

This aligns with previous reports that, in confectionery products, consumer sensory acceptance often outweighs health claims, such as “sugar-reduced” labels.^48^ Since pleasure is a primary driver for confectionery consumption, even in health-oriented products,^28^ taste optimization remains crucial. Therefore, balancing sweetness reduction with sensory appeal is essential for successful product reformulation.^5,49^

In terms of structure, the sample containing 35.0% wheat flour and 8.895% RD received the highest texture score (7.3), whereas the lowest score (5.6) was given to the formulation with 31.042% wheat flour and 10.739% RD (Table 4). These results indicate that the wheat flour to RD ratio is a key factor in textural acceptance, where increased RD may compromise structure, likely due to its hygroscopic nature or impact on gel matrix formation. Furthermore, a trade-off was observed between hardness and sensory appeal, where higher hardness correlated with lower acceptability scores. The most preferred overall sample was the sugar-reduced formulation consisting of 17.435% sucrose, 6.207% RD, and 31.358% wheat flour, suggesting that moderate reductions in sucrose, balanced with optimized flour and RD levels, can yield products with acceptable sensory qualities. These findings support the idea that effective formulation must consider not only nutritional improvements but also the impact of ingredient modifications on texture and consumer preference, factors that are critical for market acceptance.

### 3.6 Validation of Optimization Process

Design Expert software was used to generate an optimal composition for the selected parameters to minimize sucrose content and maximize RD and wheat flour in studied ranges. The optimum formulation, which achieved a desirability value of 0.87, was designed with a composition including 15.0 g/100 g sucrose, 7.49 g/100 g RD, and 32.51 g/100 g wheat flour. The determined optimum level is also compliant with the GRAS status of RD.^16^ This not only represents an advantage for the practical application of the study’s findings but also can be considered a positive outcome given the relatively high cost associated with the component.

For the optimum formulation, predicted L*, a*, b*, water activity, pH, total soluble solids, hardness, chewiness, taste, sensorial chewiness, and general acceptability values were determined to be 38.21, 12.52, −0.10, 0.60, 2.98, 86.50, 2878.5 g, 961.6 g, 6.74, 6.25, and 6.67, respectively. To determine the validity of the models, the experimental and predicted values were compared, and the results are given in Table 5. Also, these properties were determined for a control sample produced by using a conventional licorice candy formulation (Table 5). While the present study successfully formulated a sugar-reduced licorice-type extruded soft candy by incorporating RD, further research is warranted to assess the long-term stability and potential health benefits of the developed product. The integration of RD into the diet appears beneficial for managing glycemic responses, improving gut microbiota composition, and regulating body weight and inflammatory markers. Its consumption can positively modify the gut environment and is associated with a reduction in metabolic disorders, positioning RD as a promising dietary approach to support metabolic health.^50–53^ Therefore, our formulated product can go through clinical tests, and the shelf life analyses of the product can also be explored.

**Table 5:** Predicted and Experimental Properties of Samples and Main Characteristic Properties of the Control Sample

Sample	Sucrose (g/100 g)	Resistant Dextrin (RD) (g/100 g)	Wheat Flour (g/100 g)	Brix	Water Activity (*a*_w_)	pH	L*	a*	b*	Hardness (g)	Chewiness (g)	Taste	Structure	General Acceptability
Control	24.20	0.00	30.80	87.3	0.60	2.78	35.35	18.94	3.20	1477.0	162.00	7.80	7.50	7.80
Predicted	15.00	7.49	32.51	86.5	0.60	2.98	38.21	12.52	–0.10	2878.5	961.6	6.74	6.25	6.67
Experimental	15.00	7.49	32.51	86.5	0.61	2.83	36.42	17.80	3.26	1588.0	246.4	7.00	7.20	7.30

## 4 Conclusion

Licorice candies have unique characteristics, primarily due to their gelling agents and processing methods. During this study, the compositions of commercial licorice-type soft candies were determined. Also, sugar-reduced licorice-type extruded soft candies were meticulously formulated, scrutinizing the influence of three distinct ingredients. Leveraging the capabilities of Design Expert, an optimal composition emerged, featuring 15.00 g/100 g sucrose, 7.49 g/100 g resistant dextrin (RD), and 32.51 g/100 g wheat flour. The incorporation of RD proves to be a strategic addition, offering a compelling alternative that not only reduces sugar content but also fortifies the candy with dietary fibers, culminating in an impressive 7.08 g/100 g of the final product. Comparing the values of pH (2.83), total soluble solids (86.5), *a*_w_ (0.606), hardness (1588 g), chewiness (246.4), L* (36.415), a* (17.798), and b* (3.262) for the optimized sugar-reduced candy with those of the control (pH 2.78, total soluble solids 87.34, *a*_w_ 0.596, hardness 1477 g, chewiness 162.00, L* 35.35, a* 18.94, and b* 3.200), the results indicate that adding RD and reducing sucrose to these levels is feasible. Sensory evaluation showed that the sugar-reduced licorice-type extruded soft candies were well-received by consumers, demonstrating favorable ratings for taste, texture, and overall acceptability. This study presents a novel approach to formulating a sugar-reduced licorice-type extruded soft candy through the incorporation of resistant dextrin as a functional dietary fiber source. Unlike traditional licorice confections, which are typically high in added sugars and low in nutritional value, the developed product successfully achieves a significant reduction in sugar content (from 52.8 to 40.5 g) while simultaneously increasing dietary fiber content from 0 to 7.1 g per 100 g. Based on RSM statistical analysis, results (*p* < 0.05) indicated that pH, TSS, as well as textural and sensory properties were significantly affected. Therefore, the replacement of wheat flour with RD and its proportion emerged as a critical factor. The findings contribute to the growing body of knowledge on health-oriented confectionery development and provide a practical framework for integrating functional ingredients into traditional candy formulations without compromising consumer acceptability. One of the limitations of our study is the absence of a shelf life analysis. In future studies, conducting shelf life evaluations will be essential, particularly to assess the effects of independent variables over time and to validate the stability of formulations with optimized compositions. Moreover, enhancing the taste with flavor and other natural sweeteners will improve the product acceptability by consumers. In further studies, sugar-reduced licorice-type extruded soft candies can be investigated by using simulated *in vitro* and *in vivo* digestion studies to determine the possible health effects and sugar release.

## Supplementary Material



## Data Availability

The data that support the findings of this study are available throughout the manuscript.

## Notes

Sugar profile HPLC analysis calibration curves (PDF)
