# Supplementary figures and images for: Composition of Commercial Licorice Candies and Development of Sugar-Reduced Licorice-Type Extruded Soft Candy Using Resistant Dextrin
Source: ACS Omega. 2025 Jul 11;10(28):30616–26. doi: 10.1021/acsomega.5c02581 (PMC12290664; doi:10.1021/acsomega.5c02581)

1

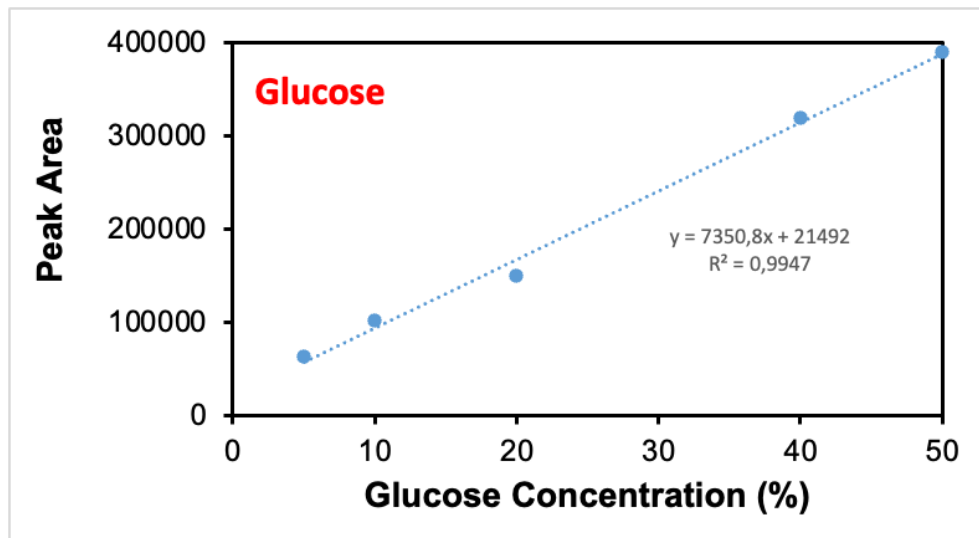

2

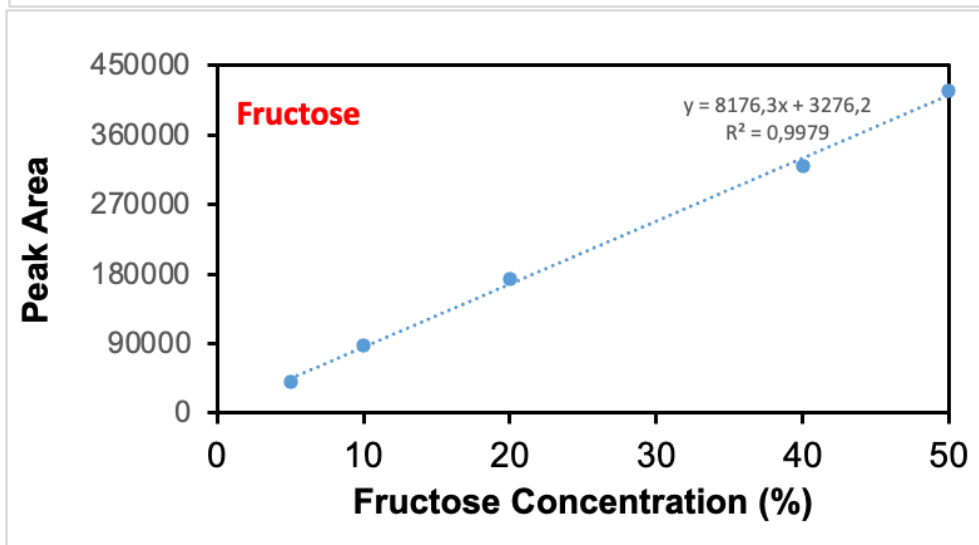

3

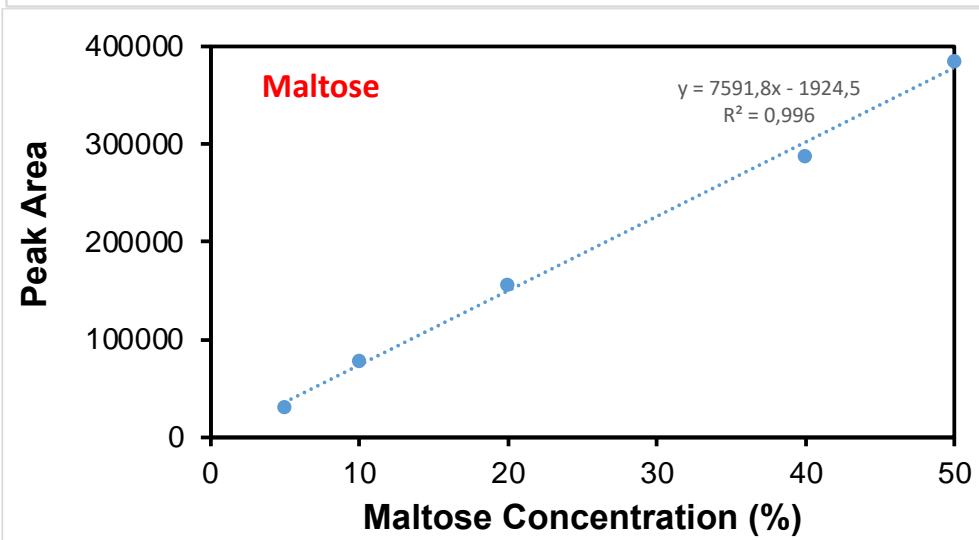

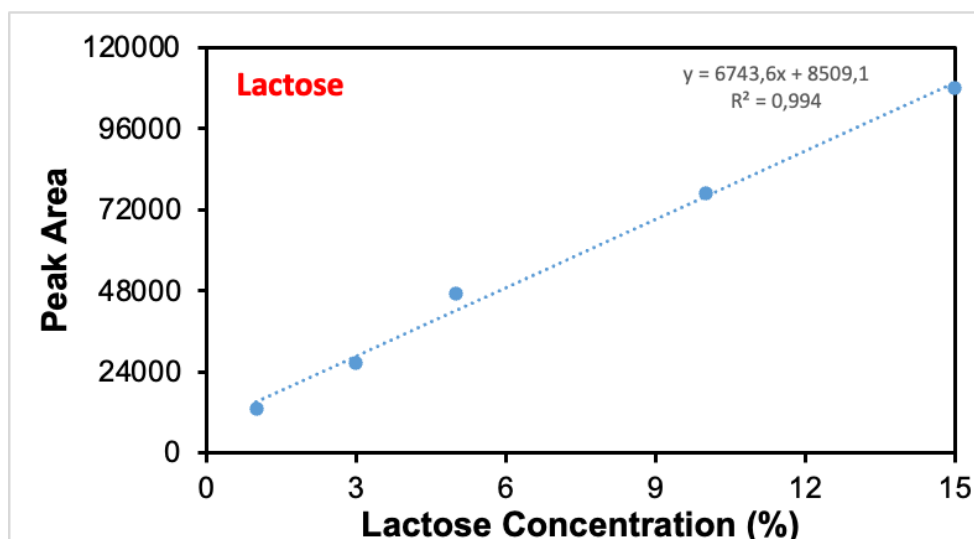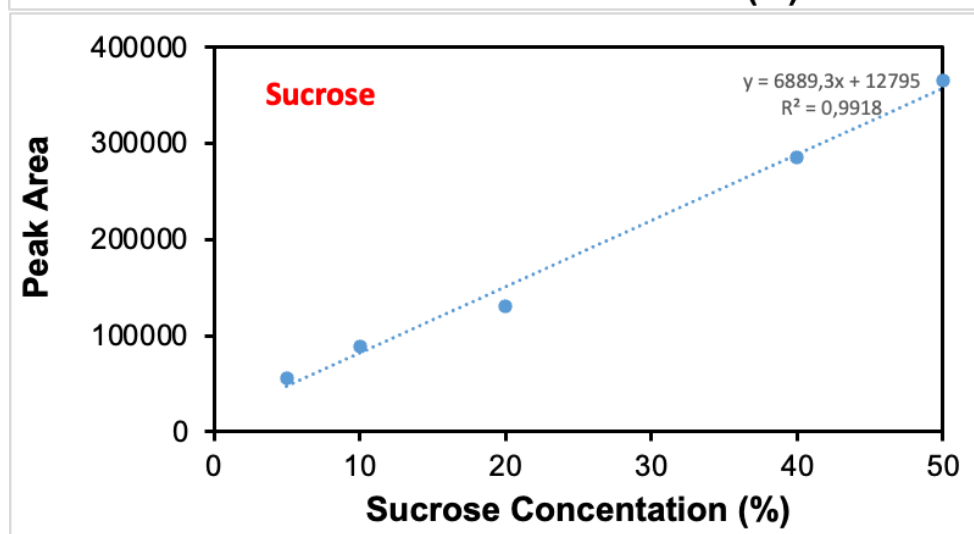

**Supplementary File 1.** Sugar profile HPLC analysis calibration curves

Supplement: Supplementary file 1 [file ao5c02581_si_001.pdf]
